# Microwave Flow Cytometric Detection and Differentiation of *Escherichia coli*

**DOI:** 10.3390/s24092870

**Published:** 2024-04-30

**Authors:** Neelima Dahal, Caroline Peak, Carl Ehrett, Jeffrey Osterberg, Min Cao, Ralu Divan, Pingshan Wang

**Affiliations:** 1 Holcombe Department of Electrical and Computer Engineering, Clemson University, Clemson, SC 29634, USA; ndahal@clemson.edu (N.D.); jeff_osterberg@hotmail.com (J.O.); 2 Department of Bioengineering, Clemson University, Clemson, SC 29634, USA; cpeak@clemson.edu; 3 Watt Family Innovation Center, Clemson University, Clemson, SC 29634, USA; cehrett@clemson.edu; 4 Department of Biological Sciences, Clemson University, Clemson, SC 29634, USA; mcao@clemson.edu; 5 Argonne National Laboratory, Chicago, IL 60439, USA; divan@anl.gov

**Keywords:** microwave sensing, flow cytometry, bacteria detection, *Escherichia coli*, sensitivity

## Abstract

Label-free measurement and analysis of single bacterial cells are essential for food safety monitoring and microbial disease diagnosis. We report a microwave flow cytometric sensor with a microstrip sensing device with reduced channel height for bacterial cell measurement. *Escherichia coli* B and *Escherichia coli* K-12 were measured with the sensor at frequencies between 500 MHz and 8 GHz. The results show microwave properties of *E. coli* cells are frequency-dependent. A LightGBM model was developed to classify cell types at a high accuracy of 0.96 at 1 GHz. Thus, the sensor provides a promising label-free method to rapidly detect and differentiate bacterial cells. Nevertheless, the method needs to be further developed by comprehensively measuring different types of cells and demonstrating accurate cell classification with improved machine-learning techniques.

## 1. Introduction

*Escherichia coli* is a type of bacteria found in the gut/intestines of animals. They are also found in the environment and foods. While most types of *E. coli* are harmless, some strains cause illnesses such as food poisoning, pneumonia, urinary tract infection, and acute kidney failure [1] in addition to the well-known, deadly sepsis [2,3]. As one of the top five pathogens contributing to foodborne illness in the United States [4], *E. coli* is estimated to cause 2138 cases of hospitalization annually [5]. Therefore, real-time, in situ measurement and analysis of *E. coli*, among other microorganisms, are important.

Conventional culture-based methods remain the gold standard for bacteria detection and identification, but the methods are time-consuming. Various culture-independent methods have been developed for rapid sample-to-answer operation. Nucleic acid-based approach can offer high sensitivity and specificity, but its procedure is complex with steps involving DNA extraction and purification, polymerase chain reaction (PCR), and detection [6]. An immunolabeling method, such as reported in [7] can detect *E. coli* but requires a labeling reagent with limited shelf life. Label-free Raman spectroscopy reported the detection and identification of single *E. coli* isolates, but background noise interference and a complicated system are some of the issues [8]. W. Wu et al. reported on a chemiluminescence-based method using a digital microwell array chip for single *E. coli* cell detection. However, the method required a 2.5-hour-long incubation period during which hydrolysis of bacterial cells occurred [9]. The entire process could take up to 4 h, which is an improvement over the conventional culture method but is still time-consuming, nonetheless. B. Thakur et al. reported on a graphene-based field-effect transistor to detect *E. coli*. However, a single cell was not detected although potential to do so was mentioned [10]. 

Impedance cytometry can provide a label-free technique to measure the electrical properties of a single cell, and to differentiate sub-populations [11,12,13,14]. A multifrequency impedance measurement approach is reported in [11] where conductivity and permittivity of red blood cells and red cell ghosts were measured, and the two different cell populations were differentiated. A high throughput of 200 cells/second for single-cell spectroscopy was reported. However, for single cell characterization using the model developed in [11], the orientation for each cell would need to be known which adds complexity to the method when cell shape is not spherical as in the case of *E. coli* cells. One of the major limitations of impedance flow cytometry method is the variation of the impedance signal with the position of the cell in the channel [15]. In [16], a device with a coplanar differential electrode was discussed which accurately resolved the spatial position of the cell. Their design included floating electrodes along with the excitation electrode and sensing electrode; the floating electrodes at each side of the excitation electrode enabled the determination of the cell spatial position. C. V. Bertelsen et al. reported the use of impedance flow cytometry for classifying viable and non-viable *E. coli* cells [17]. Based on the results in [18,19], where the authors used microwave-based methods for single cell measurement, classifying cell species or strains is more challenging than classifying viable and non-viable cells of the same species. In [20], an impedance flow cytometer was used to assess *E. coli* concentration in drinking water, and to differentiate *E. coli* from polystyrene beads. Moreover, Gram-positive bacteria *Stapylococcus aureus* was differentiated from Gram-negative bacteria *E. coli*, but measurement accuracy was not reported.

Dielectrophoresis is another method for cell/particle detection and differentiation in which neutral but polarizable particles are exposed to a non-uniform electric field [21]. Dielectrophoresis and microfluidics can be integrated for label-free characterization/differentiation of bioparticles such as normal oral epithelium and oral and oropharyngeal cancer cells in [22]. Acoustophoresis is another label-free and noncontact method of particle separation that uses acoustic fields to induce motion in the subjected particles [23,24]. In [23], dielectrophoresis and acoustophoresis methods were combined in a microfluidic chip to obtain continuous-flow label-free focusing, switching, and sorting of particles/cells. In [24], acoustophoresis was used to separate living and dead cells. Both dielectrophoresis and acoustophoresis methods exert little impact on the living cells such that their viability is generally not affected [22]. Droplet microfluidics is an emerging technology for single-cell analysis, which allows information extraction at the genomic, transcriptomic, proteomic, and metabolomic level. Moreover, a small sample size, normally in the picoliter to nanoliter range, is needed. Rapid detection is, however, a challenge because droplet microfluidics produces a large number of tiny, fast droplets [25].

Microwave-based methods have been demonstrated for single-cell measurement, such as baker’s yeast cells [18], *Candida* yeast cells [19], Chinese hamster ovary cells (CHO) [26], human monocytes THP1 cells [27], and Jurkat cells [28]. The measurements are label-free and non-invasive. Nevertheless, there is a lack of sensitivity for single *E. coli* detection [29]. A microstrip-based microwave sensor for single lymphoblast cell (C1498) detection is reported in [30]. These cells have a typical diameter of approximately 20 µm, which is several times larger compared to *E. coli* (0.5 μm × 1–2 μm). Moreover, only cell detection is achieved, and no population classification is reported [30]. Two microwave sensors (1) coplanar waveguide (CPW)-based, and (2) split-ring resonator-based for dielectric characterization of single cells were reported in [31]. Polystyrene particles of 10 µm and 20 µm diameters, and mammalian cells with a diameter of approximately 13.8 ± 3.8 µm were detected and classified. A higher sensitivity microwave sensor is needed to detect single *E. coli* cells. In this work, we developed microstrip sensing devices integrated with a microwave flow cytometer to detect single *E. coli* cells of B strain and K-12 strain. A simple machine-learning (ML) model was developed to differentiate the two strains.

## 2. Materials and Methods

### 2.1. Bacteria and Sample Preparation

Of the various *E. coli* strains, *E. coli* B and *E. coli* K-12 are the most frequently used laboratory strains, and hence were used in this study. *E. coli* B strain and *E. coli* K-12 strain were streaked from lab frozen stocks on separate fresh Luria agar plates and incubated at 37 °C until several colonies were formed in each. Then, a colony of each strain was inoculated in 3 mL of Luria broth (LB) and incubated overnight in a shaker at 37 °C and 100–125 rpm. The culture was harvested after an overnight incubation at which point the bacterial cells were in the stationary phase of growth. A fresh 1:100 dilution of overnight culture in LB was carried out and incubated in the shaker at 37 °C and 100–125 rpm for approximately 3 h to obtain bacterial cells in the logarithmic phase of growth.

Prior to injecting bacterial cells in the microwave sensor, bacterial culture was diluted in sterile de-ionized (DI) water at a ratio of 1:50. All the measurements were completed within one to two hours to ensure bacterial cells remained viable during the course of measurement.

### 2.2. Microstrip Sensing Devices and Microwave Spectroscopic Flow Cytometer

Different microwave structures, such as resonators, CPW, and microstrip line, have been used for cell sensing. Resonators yield high sensitivity but have limited operating frequency points [32]. In CPWs, signal strength varies depending on cell position relative to CPW electrode [33]. Microstrip provides higher sensitivity than CPW and is much less sensitive to cell position [18,19]. Thus, microstrip sensing devices are considered in this work since reproducible microwave signals are essential for ML analysis and classification of single *E. coli* cells.

**Figure 1:**
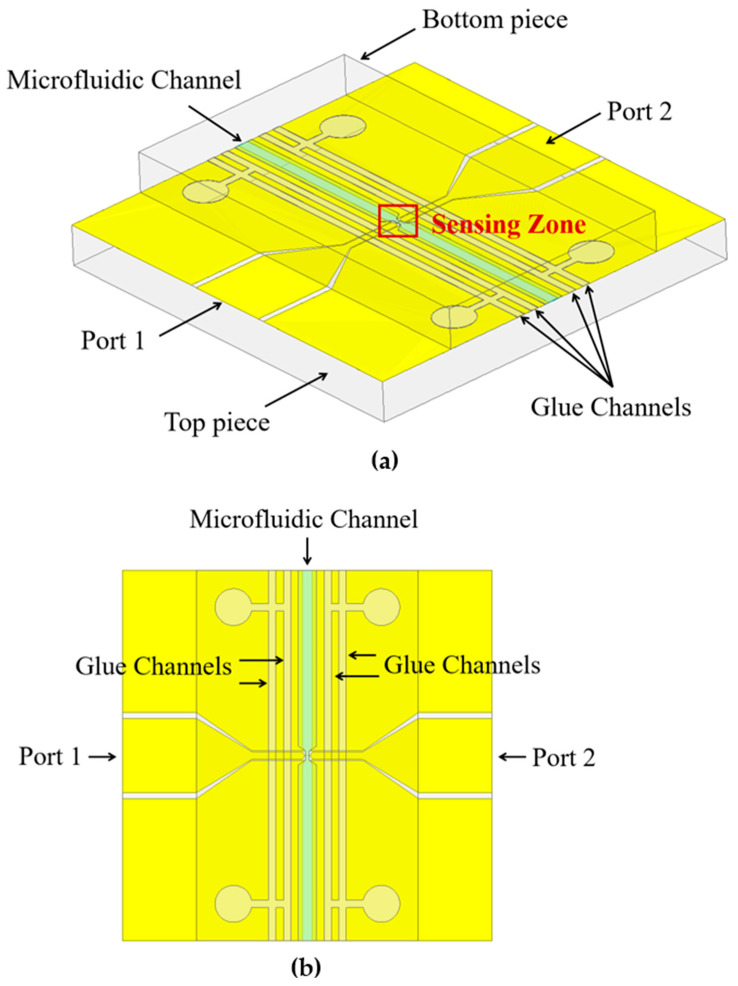
(**a**) A 3-D model of the microstrip sensor. (**b**) top view of the microstrip sensor.

We used a 10 µm wide microstrip sensing electrode, which transitions to a 2 mm wide CPW on each side to connect to a SubMiniature version A (SMA) connector. The SMA connectors were connected to a two-port Vector Network Analyzer (VNA) (Copper Mountain Technologies, Indianapolis, IN, USA) using coaxial cables. A 3-D model of the device is shown in Figure 1a and the top-view is shown in Figure 1b. The microstrip sensing electrode is in the sensing zone. The microfluidic channel runs perpendicular to the signal line, and the glue channels run parallel to the microfluidic channel.

The top and bottom pieces were fabricated on 4-inch diameter 1 mm thick borosilicate wafers. The top piece contains the CPW–microstrip–CPW signal line and CPW ground plane. The bottom piece contains the microstrip ground plane, microfluidic channel, and glue channels. When assembled, the top piece forms a cover for the microfluidic channel and the glue channels. The assembled device is 10 mm × 10 mm, and the microfluidic channel and the glue channels extend the full width of the device. The microfluidic channel tapers from 500 µm outside of the sensing zone down to 100 µm in the sensing zone. The wide part of the channel outside of the sensing zone allows microfluidic tubes to be inserted at the channel ends for samples to be injected using a syringe. Optical adhesive glue (UV glue) was manually applied to the glue channels to serve as an adhesive between the top and the bottom pieces.

**Figure 2:**
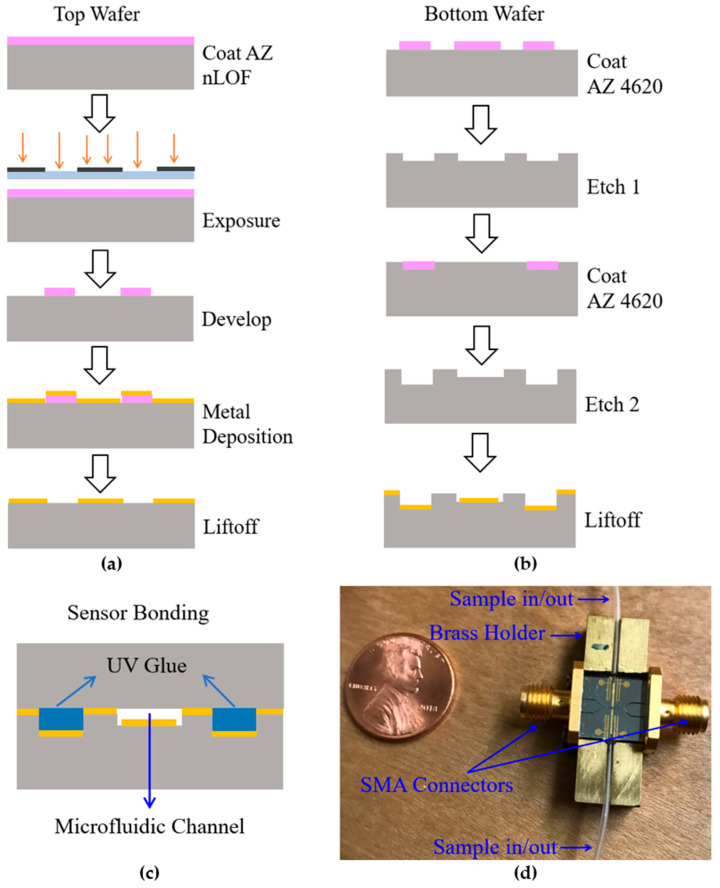
Fabrication process of bacteria sensor. (**a**) Top wafer. (**b**) Bottom wafer. (**c**) Graphical depiction of sensor bonding where top and bottom pieces are held together by UV glue. (**d**) A picture of the assembled sensor (a United States penny shown for scale).

The cleanroom fabrication of the microstrip device started with borosilicate wafers. Using a liftoff process, 20-Chromium/200-Aurum metal was patterned on the top wafer to create signal line and ground plane. The bottom wafer fabrication process included two etch steps. In the first etch, 1.5 μm deep microfluidic and glue channels were created. In the second etch, the microfluidic channel in the sensing zone was protected while etching an additional 8–10 μm in the microfluidic and glue channels. AZ nLOF and AZ 4620 were the photoresist materials used. The fabrication process is graphically shown in Figure 2. A graphical depiction of bonded sensor/device where top and bottom pieces are glued together by UV glue is shown in Figure 2c. The assembled sensor with a brass holder to facilitate SMA connection and to improve robustness of the device, and silicone tubes for sample injection is shown in Figure 2d.

**Figure 3:**
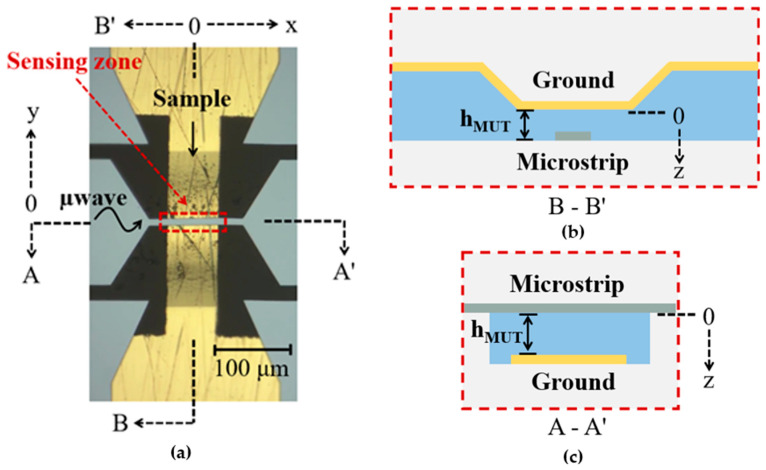
(**a**) Microscopic image of the bacteria sensing device. (**b**) Cross-section of the sensing zone along the A-A′ axes (top) and (**c**) cross-section of the sensing zone along the B-B′ axes (bottom).

Shown in Figure 3a–c are a picture of the microstrip sensing device taken under the microscope, and the cross-section schematics of the device, respectively, used for *E. coli* measurements. Channel height, *h_MUT_*, in Figure 3b is critical for single *E. coli* measurement. The microstrip devices developed in [18,19] for successful detection and differentiation of yeast species, hereon referred to as yeast device, were scaled to obtain targeted *h_MUT_* values. The yeast device consisted of a 10 μm wide microstrip with 9 μm channel height. The yeast species were from 3 to 9 µm in size [18,19], and the predicted smallest measurable polystyrene particle with the yeast device was 1.7 μm, assuming cell microwave signals correlate with cell volume linearly [18]. In comparison, *E. coli* cells are only 0.5 μm × 1–2 μm in size.

The electric field intensity, *E*, of a microstrip is inversely proportional to the microstrip height, *h_MUT_* [34]. When a cell is present in the field, it produces a microwave signal, which is determined by the microwave–cell interaction intensity, I.

(1) $$I=\frac{1}{4}(\varepsilon_{c}-\varepsilon_{m})\times V\times {\left|E\right|}^{2}$$

where *ε_c_*, *ε_m_*, and *V* are the average permittivity of the cell, the permittivity of the mediumin which the cell is suspended, and the volume of the cell, respectively. 

Rearrange Equation (1) to obtain Equation (2).

(2) $$\frac{I}{\Delta \epsilon }=\frac{1}{4}V\times {\left|E\right|}^{2}$$

Therefore, *I/*Δ*ε* for a 1.7 μm particle/cell is 2.9 × 10^−7^ V^2^⋅m. To achieve the same value of I/Δε for *E. coli*, assuming a volume of 0.5 μm × 1 μm × 0.5 μm, the maximum electric field magnitude needed is 2.2 × 10^6^ V/m. This corresponds to a channel height of approximately 2.25 μm. However, cell sizes have a large variation. Hence, an even larger electric field magnitude may be needed to detect all *E. coli* cells in a cell suspension. 

Moreover, reducing the channel height to <2.25 μm in a practical device is extremely challenging. The microfluidic channel is 500 μm wide and 10 mm long. In the sensing zone, which is 250 μm long, the width of the channel is 100 μm. A pressure of 3.5 × 10^4^ mbar is needed to pump DI water at a flow rate of 10 µL/min, which makes the sensor impractical for a rapid and high-throughput cell measurement. As a result, a multi-section microfluidic channel which is wider and taller everywhere except in the sensing zone was built. The channel height is approximately 1.5 μm in the sensing zone and approximately 10 μm elsewhere. In the microscopic image of the microstrip sensor shown in Figure 3a, a shadowed region, which is the narrow sensing zone, is seen. A channel height that tapers (due to isotropic wet etch) from 10 μm to 1.5 μm is seen in the sensing zone along the B-B′ axis as shown in Figure 3b. The cross-section of the sensing zone along the A-A′ axis is shown in Figure 3c.

**Figure 4:**
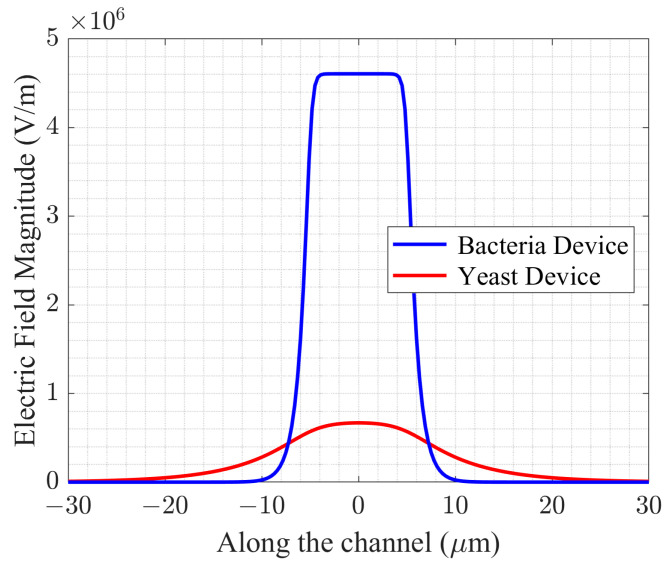
Simulated electric field magnitude of the bacteria device and the yeast device.

Ansys HFSS 2021 R1 (High Frequency Structure Simulation Software) simulations comparing the electric field magnitudes of the bacteria device (1.5 μm tall channel) and the yeast device (9 μm tall channel) is shown in Figure 4. The *x*-axis labeled “Along the channel (μm)” is on the B-B′ axes. The center of the sensing zone is at 0, and ±30 μm are on either side of the center. The maximum electric field magnitude of the bacteria device is 4.5 times larger than that of the yeast device and enables detection of *E. coli* single cell. 

**Figure 5:**
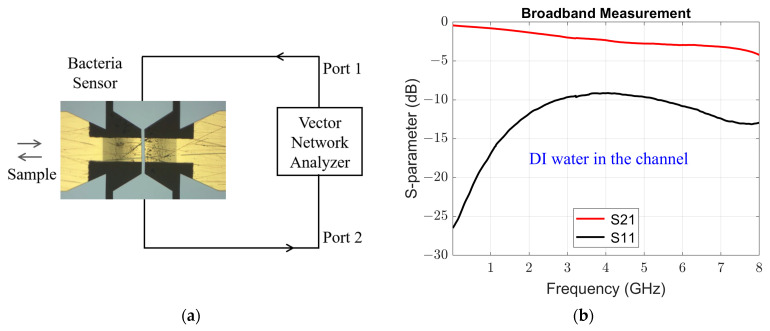
(**a**) Test setup using a vector network analyzer (VNA) and bacteria sensing device. In (**b**) graph shows broadband scattering parameters, insertion loss (S21) and return loss (S11), measurement with de-ionized (DI) water in the sensing zone.

The test setup is illustrated in Figure 5a. A two-port VNA was connected to the bacteria sensing device using coaxial cables to measure scattering parameters. The bacterial cells suspended in DI water can be injected in/out of either side of the microfluidic channel. The broadband measurement with DI water in the sensing zone is shown in Figure 3b. At 1 GHz, an insertion loss (S21) of −0.8 dB and a return loss (S11) of −17 dB was observed. 

## 3. Results

**Figure 6:**
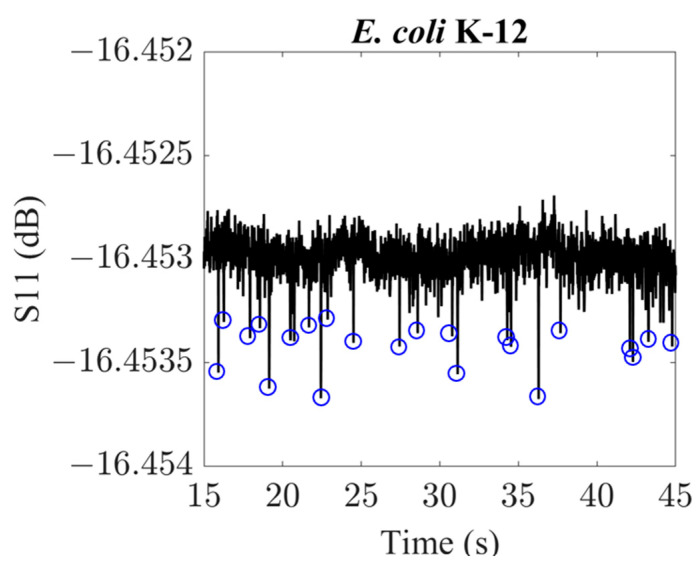
Raw S11 (dB) measurement of *E. coli* K-12 using the bacteria sensor. Each spike in the measurement, such as circled in blue, is due to an *E. coli* K-12 cell passing the sensing zone. Spikes are identified as points at which, after the measurement data is detrended and standardized, the Mahalanobis distance of the four-dimensional measurement exceed three standard deviations from the mean.

*E. coli* B strain and *E. coli* K-12 strain were measured at various frequency points ranging from 500 MHz to 8 GHz. A typical S-parameter (S11 magnitude) measurement of *E. coli* K-12 strain at 1 GHz is shown in Figure 6. Each spike in the measurement, for example circled in blue below, corresponds to an *E. coli* cell passing the sensing zone verified by simultaneous video recording. The video recording can be found under the Supplementary Materials section.

**Figure 7:**
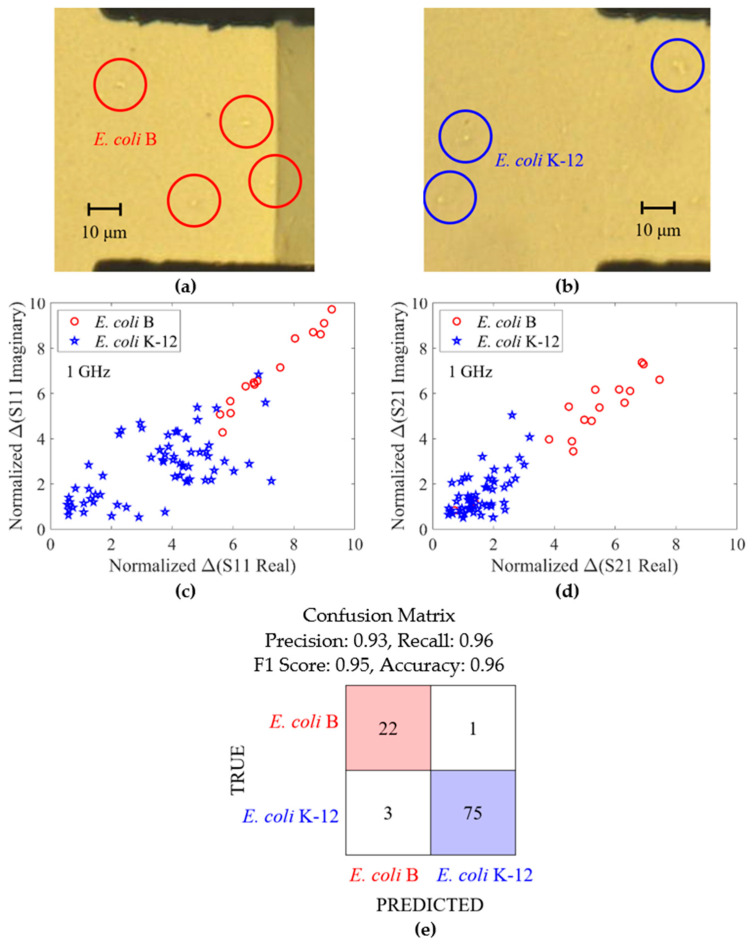
Microscope images of (**a**) *E. coli* B strain and (**b**) *E. coli* K-12 strain in the bacteria sensor. The cells look visually indistinguishable. (**c**) Peak standardized detrended Δ(S11 real) versus peak standardized detrended Δ(S11 imaginary). (**d**) Peak standardized detrended Δ(S21 real) versus peak standardized detrended Δ(S21 imaginary). (**e**) Confusion matrix resulting from a LightGBM classification algorithm. Each of the two cell strains are observed in a separate experiment. The resulting peak standardized detrended signal values are a four-dimensional vector for each cell, composed of real and imaginary ΔS11 and ΔS21 values. These are used as the regressors for the LightGBM classifier, with the classification target being a binary indicator of cell strain (*E. coli* B or *E. coli* K-12). Five-fold cross-validation is used to ensure the classifier predictions reported in this confusion matrix describe the classifier performance on data points that are not included in the training data used to fit the model.

The microscope images of *E. coli* B strain and *E. coli* K-12 strain are shown in Figure 7a,b, respectively. Visually, the bacterial cells look identical, and hence cannot be differentiated. Shown in Figure 7c,d are peak standardized detrended ΔS-parameter real versus peak standardized detrended ΔS-parameter imaginary for S11 and S21, respectively. To produce these values, the raw measurements were first detrended using a Gaussian filter on each of the four measurement components, with a standard deviation of 20. The four detrended measurement components were then standardized to have mean zero and standard deviation 1. Peaks were identified in the resulting four-dimensional detrended standardized measurement as points at which the Mahalanobis distance of the measurement from the mean exceeded three standard deviations. The peak standardized detrended (hereon referred to as “normalized”) ΔS-parameter is calculated as the raw detrended S-parameter signal minus the mean detrended S-parameter signal, divided by the standard deviation of the detrended S-parameter signal.

Microwave measurement at 1 GHz shows a clear separation between the two strains of *E. coli*. The between group heterogeneity of the peak standardized detrended ΔS values is shown by fitting a LightGBM classification model to predict cell strain from these values. The LightGBM classifier was selected since it achieved higher accuracy than other algorithms tested with this data (Support Vector Machines, Multi-layer Perceptrons, Quadratic Discriminant Analysis, and Random Forests). After LightGBM was chosen due to its performance with default settings (learning rate 0.1, no max tree depth, minimum 20 child samples in each node, 100 trees), we carried out optimization of the classifier’s setting using the Optuna library [35], but the default settings were found to be the most performant. The model’s performance was evaluated using five-fold cross-validation, i.e., the model was trained five times, each on 4/5 of the full data, and evaluated on the remaining 1/5 of the data. This ensures the model’s performance is not inflated by being evaluated on data included in its training regime. The resulting confusion matrix, shown in Figure 7e, shows the model’s predicted classification of each cell, when that cell is not included in the training data of the model. A high accuracy of 0.96 was obtained, demonstrating the normalized ΔS values carry sufficient between-group heterogeneity to support reliable machine-learning classification of these cell strains. 

**Figure 8:**
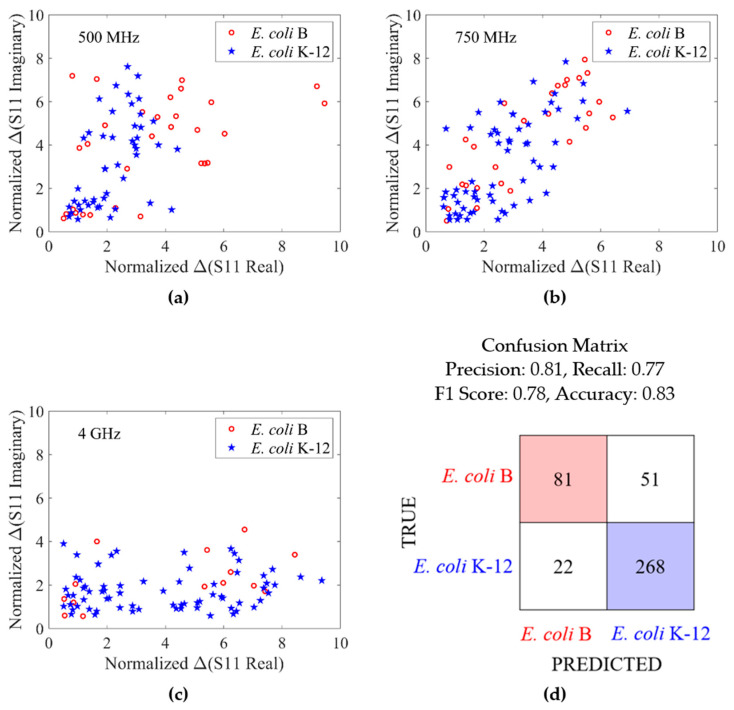
Measurement of *E. coli* B and *E. coli* K-12 strains at 500 MHz, 750 MHz, and 4 GHz. Peak standardized detrended Δ(S11 real) versus peak standardized detrended Δ(S11 imaginary) at (**a**) 500 MHz, (**b**) 750 MHz’s, and (**c**) 4 GHz. (**d**) Confusion matrix resulting from multi-layer perceptron method.

As stated earlier, *E. coli* B and *E. coli* K-12 strains were measured at multiple frequency points. In Figure 7, data measured at 1 GHz, which provided the best detection accuracy was shown. In addition, the normalized ΔS11 of the two *E. coli* strains measured at 500 MHz, 750 MHz, and 4 GHz are shown in Figure 8a–c. Each cell measured was observed at only one of the four frequency points. At these three frequency points, data overlap is seen. A multi-layer perceptron (MLP) method was also explored for species differentiation. A single hidden layer of 100 neurons was used. Despite the fact that the task of cell differentiation is appreciably more challenging in the context of this dataset than the one shown in Figure 7, the MLP model obtains an accuracy of 0.83, as shown in the confusion matrix in Figure 8d. While this is lower than the 0.96 accuracy obtained at 1 GHz, it shows the great potential of ML method at differentiating cells species/strains when visually the data sets look very similar. Thus, the ML method can be employed when the best frequency point for species/strain differentiation is not known.

**Figure 9:**
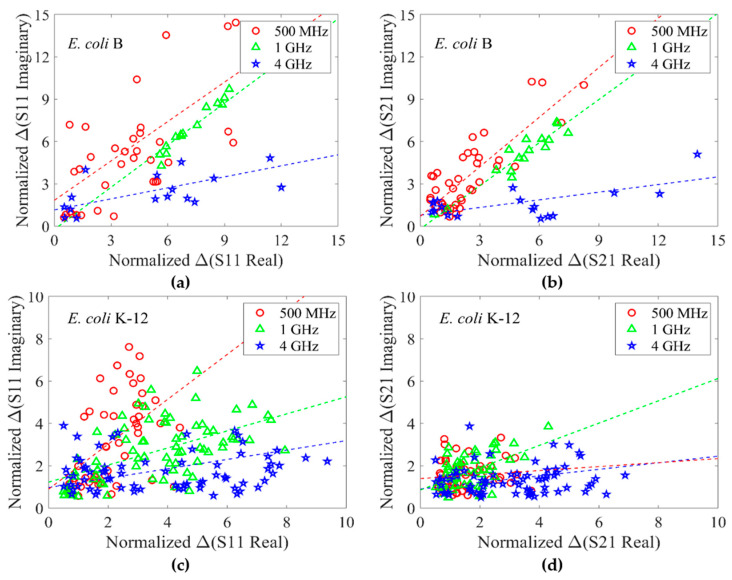
*E. coli* B and K-12 strain measured at multiple frequency points. (**a**) Normalized Δ(S11 real) versus normalized Δ(S11 imaginary) for *E. coli* B strain. (**b**) Normalized Δ(S21 real) versus normalized Δ(S21 imaginary) for *E. coli* B strain. (**c**) Normalized Δ(S11 real) versus normalized Δ(S11 imaginary) for *E. coli* K-12 strain. (**d**) Normalized Δ(S21 real) versus normalized Δ(S21 imaginary) for *E. coli* K-12 strain.

Shown in Figure 9a,b are normalized ΔS-parameter real versus normalized ΔS-parameter imaginary for S11 and S21, respectively, for *E. coli* B strain measured at 500 MHz, 1 GHz, and 4 GHz. Similarly, shown in Figure 9c,d are measurement data for *E. coli* K-12 strain. Each cell is only measured once at one frequency point. In general, S11 measurement for both the strains shows a similar frequency trend where the slope of the linear regression line (shown using the dashed line) decreases as the frequency increases. This trend is only partially present in Figure 9a,d.

At lower frequency points, Δ(S-param imaginary) is more significant than at higher frequency points. This is due to the cell membrane having a larger effect at lower frequency points (β-dispersion). As the frequency increases, the impact of cell membrane on microwave measurement is reduced. The differences between the permittivity of cell cytoplasm and the surrounding medium (1:50 ratio of LB media and DI water) has a more significant effect at higher frequencies [36,37,38]. 

## 4. Discussions and Conclusions

The obtained results show the microwave flow cytometry approach is viable for single bacterial cell detection. Compared with prior state of the art [28], the demonstrated sensitivity is a significant improvement. The results also show a new path for reproducible measurements of bacterial cells in flow at tens of cells per second. The obtained cell properties or signals enable ML-based cell feature extraction and differentiation. Nevertheless, the reduced channel height significantly limits measurement sample flow rate in volume per unit time. At the same time, Equations (1) and (2) imply potentially reduced range of measurable particle sizes since signal-to-noise ratio will decrease with channel height. The reduced channel height in the sensing zone also poses another challenge in which the bacterial cells stick to the channel surface and to each other over the course of a few hours and clog the device, rendering it useless. Attempts to dislodge the cells using surfactants and solvents have not proved to be successful.

In summary, reducing microstrip line channel height is shown as an effective approach to increase sensitivity while preserving measurement reproducibility for single bacterial cell studies. The obtained results of *E. coli* B and *E. coli* K-12 between 500 MHz and 8 GHz show cell properties are frequency-dependent and strain-dependent. Preliminary machine-learning analysis shows accurate cell strain determination. Nevertheless, a significant number of bacterial cells of different types and under different growth conditions need to be measured at different frequencies and guided by an established protocol. Improved ML techniques need to be developed to demonstrate accurate identification of cell types. Effective device cleaning techniques and methods are needed to increase the life of the device such that all cell types can be measured using only one device for the most accurate comparison.

## Data Availability

The data is not located in a publicly archived dataset yet as it is an ongoing research.

## Supplementary Materials

The following supporting information can be downloaded at: https://www.mdpi.com/article/10.3390/s24092870/s1.
